# Supplementary material for: A recurrent neural network framework for flexible and adaptive decision making based on sequence learning
Source: PLoS Comput Biol. 2020 Nov 3;16(11):e1008342. doi: 10.1371/journal.pcbi.1008342 (PMC7673505; doi:10.1371/journal.pcbi.1008342)
Supplement: Attachment — Submitted filename: Response to reviewers.docx [file pcbi.1008342.s001.docx]

Responses to Reviewer's Questions

Here, please find our responses to the reviewers. We are grateful for many valuable suggestions, which we have incorporated into our revision. The notable changes in the revised paper are as follows.

1. We revised the Methods and Results and provided further details of the framework.
2. We have revised the discussion on reinforcement learning to clarify potential confusions.
3. We added a new section in Discussion for experimentally testable predictions based on our framework.
4. In the reply, we have provided new figures and analyses to support our *when/which* analyses. We, however, feel that these analyses may be too detailed for the general readership. Given the current length of the manuscript, we opted not to include them in the manuscript, but would be happy to oblige if the reviewers feel that they are necessary.

With all these changes, we hope the reviewers agree that the result is a better paper. Below, the referees' comments are quoted in black; our responses are in blue.

**Comments to the Authors:
Please note here if the review is uploaded as an attachment.**

Reviewer #1: This manuscript describes a RNN based on GRU units, which is trained to solve a fairly complex sequential probabilistic task. Specifically a sequence composed of an alphabet of 10 symbols (each of which is probabilistically associated with a correct Left/Right response) is presented until a choice is made. The optimal strategy is to integrate information across symbols until enough evidence for a decision is available. As the authors point out this task has similarities with natural language processing, thus the use of a GRU. After training the RNN solves the tasks across nontrained sequences. The units of the RNNs seem to qualitatively resemble the behavior of neurons recorded during animal experiments using similar tasks, including response, log likelihood and "urgency" neurons. The analyses and simulations seem well executed, however a weakness is the conclusions in terms of how the results improve our understanding of brain function. Some reference is made regarding capturing the properties of basal ganglia, but there is no direct clear link.

1.1 Can the authors make any clear cut experimental predictions? For example are the authors predicting that animals are attempting to predict both the subsequent stimuli and reward with equal weighting (as implemented by their loss function)?

The *when/which* analyses in the Results are basically all experimental predictions, including their network connection patterns, the dissociable manipulation effects, etc. In the discussion, we proposed that our model may describe the computation in the basal ganglia. Therefore, one may record from the basal ganglia and study whether basal ganglia neurons exhibit properties of *when/which* units in the model.

We have now added a new section in Discussion to discuss potential experiments that can be done to test our framework (Line 850-868):

Experimental testing

Many of the predictions based on our framework can be tested experimentally. For example, we have discussed the when/which units in Task 1. One can directly record neurons from a brain area related to evidence accumulation, e.g. the basal ganglia, to see whether they may be sorted into two similar categories and contribute to decision making in a manner consistent with our model. Furthermore, one can look at such brain areas to see whether there is a representation of combined predictions of sensory, motor, and reward events during decision making as the framework indicates. The gating mechanism in our framework may have its correspondence in these brain areas, e.g., the direct and indirect pathways in the basal ganglia. One can test whether the model could predict the consequences of experimental manipulations of the gating mechanism. The difference between our model and standard RL models is also interesting. For example, in the two-step task, our model’s performance depends on the network’s ability to maintain history information across trials through its response dynamics. Therefore, we predict that disturbance during inter-trial intervals may disrupt the performance. This should not be expected if the brain uses a more RL-like strategy, in which the learning is based on synaptic plasticity instead of network dynamics.

1.2 The authors attempt to make a link to the biology, and make a few general statements about similarities to the basal ganglia, and a link between GRUs and the basal ganglia circuitry. This seems counterintuitive to me for a number of reasons including the fact that the basal ganglia circuitry is all inhibitory. In regards to the potential biological relevance it would be important to determine if gated units (which can have infinite long memory) are needed. Indeed, since no parametric studies are presented it would be helpful to do so, and one way this could be accomplished is to compare GRU performance with ReLU performance. That is, is this a task that requires the essentially infinite long-term memory of gated units, or can it be achieved equally well by more realistic ReLUs?

In the current study, the link between GRUs and the basal ganglia circuitry is only at the conceptual level and is based on the gating structure shared by both. We recognize that the current framework is very simplified, and that the network details may be quite different from that of the actual brain network, including the basal ganglia. But the current manuscript is intended to serve only as the beginning of a new approach to understand the decision making circuitry in the brain. Therefore, we have not included many explorations regarding network details in this manuscript. These are being or will be investigated in our lab. We actually recently published another paper in which we built a network model based on the same conceptual framework, but the model was constructed to reflect the basal ganglia anatomy, including the inhibitory projections and direct/indirect pathway circuitry. We showed that this conceptual framework does work. (Chen, X., Yang, T. A neural network model of basal ganglia' decision-making circuitry. *Cogn Neurodyn* (2020).)

Also for the same reason, we are not aiming to compare the GRU network against the others. GRU networks can be considered as a variant of RNN in general. It is conceivable that we can obtain similar results by training an RNN. Whether the structure of the resulting network resembles our GRU would be interesting to explore. We choose GRU networks because they have been shown to be adept at processing sequences, and they have been successfully used in the NLP field. We are building on these results to ask whether the computation in the brain resembles sequence learning.

1.3 Does performance exhibit a commutative property. That is, is the Reaction time the same for shapes 1,2,3 and 3,2,1 (controlling for cases in one shape basically solves the task)? This is an important question because from a mathematical stance it should be the case, but biologically speaking it is probably not the case, because animals are generally heavily biased by recency effects. Relatedly is the model equally weighing all evidence or preferentially weighing more recent evidence (i.e., forgetting early evidence)?

In the current version of the task, the evidence is accumulated by the network model without leak and the performance shows a commutative property. In Figure 3d, we show that the first shapes, middle shapes, and the late shapes affect the network choice equally.

Recency effects are less common in this type of accumulation tasks. In both the probabilistic reasoning task (Kira, Yang & Shadlen, 2015) and the random dots task (Kiani, Hanks & Shadlen, 2008), people did not report a recency effect.

In fact, whether our network shows a commutative property depends on the training set. If we used a training set with a recency effect, the network would exhibit such an effect. The recency effect is part of the statistical structure that the model can learn through training.

1.4 Something that is a bit glossed over in the presentation is that the network is not actually making a decision, there seems to be a postprocessing stage in which information from the network is analyzed to see if a criteria is reached and then the simulation is terminated. This is a bit misleading as many people will be left with the impression the RNN is autonomously making a reaction time decision. Thus, this postprocessing stage should be strongly emphasized in the results.

There is actually no postprocessing stage in the network. The network choice is based on a softmax choice based on all output units' activities. The network makes reaction-time decisions autonomously during the testing sessions.

The confusion may be due to how we described the training dataset. The choices in the training dataset are indeed generated with a DDM-like mechanism, which has a pre-determined decision bound that controls the speed-accuracy balance. The choices during the testing phase, however, are fully generated by the network itself.

We have revised our manuscript to make this point clearer (Line 291-298).

1.5 Apparently, the model was trained with Adam, but absolutely no information is given about this critical component of any model.

We have added the details of Adam in the manuscript (Line 218-228).

1.6 It would be helpful to show some raw data of model performance as is often shown for standard RNN models during the performance of the task.

We are not sure what kind of raw data the reviewer is suggesting exactly. Here, we include a new supplementary figure showing how the network’s prediction correct rate of all events and the loss change during the training of Task 1.

Fig Supp1. Model performance. a. The overall prediction correct rate during the training session. The star indicates the correct rate in the test set. b. The loss decays with training. The star indicates the loss in the test set. The width of the lines indicates the SE across runs.

1.7 The performance measure does not allow the reader to understand how well the model is integrating, how well can it perform just looking at the last N shapes?

In Figure 3d, we show that the early shapes and the late shapes affect the choice equally, which indicates that the integration is nearly perfect.

1.8 Line 42. "framework".

Fixed

1.9 Line 92. "ranging"

Fixed

1.10 Li 280. Just "Performance" (behavior gives the impression there were animal studies).

Fixed

1.11 Li 735 "reported THAT lesions"

Fixed

Reviewer #2: In the manuscript titled "A Recurrent Neural Network Framework for Flexible and Adaptive Decision Making based on Sequence Learning", Zhang and Colleagues used natural language processing (NLP) framework and trained a network of gated recurrent units in four different experimental setting. Specifically, similar to networks in the NLP that are trained to predict text sequences, authors trained their networks to take inputs in the form of event sequences (sensory and reward outcome events) and predict future events through supervised learning. Networks were trained on a probabilistic reasoning task, a multi-sensory integration task, a confidence/post-decision wagering task, and a two-step task. Authors showed that networks can learn perform the task and showed behavior similar to that observed to animals. Additionally, authors found units that resembled activity of recorded neurons in different areas of the brain.

Overall, I found the paper suitable for publication in journal of PCB and their proposed framework interesting. However, some details of training procedure and analysis require further justification. Moreover, strong conclusions are drawn without enough supporting information, which needs to be addressed. Please find my comments below:

Major concerns:

2.1 1) Page 14, lines 267-270: Why a drift-diffusion model (DDM) with collapsing boundary was used to simulate the choice used for training the network? It is sounds circular to train the network with a certain model and observe units that resemble the key parts of that model. How much of these observations depend on the model behind choice behavior? Why not train the network with the optimal algorithm? Authors need to clarify this.

We can certainly use a DDM with constant bounds, but by using collapsing bounds, we can increase the complexity of the task and further demonstrate the network models' ability to learn complex temporal contingencies.

We do not agree that our logic is circular. The network is not designed for this particular task or for implementing this particular model. There are no explicit mechanisms in the network model to accumulate the information or to use a bound for decision making. The network has to learn to behave so based on the training sequences. Training the model to reproduce the choice and reaction-time behavior does not necessarily lead to the units that we describe in the manuscript. For example, the attractor network model proposed by Xiao-Jing Wang (Neuron, 2002) could exhibit similar behavior but showed distinct response patterns. For example, the attractor network state reaches a fixed point at the time of decision. There are no dissociable *when/which* units that may support speed-accuracy trade-off.

It has been shown that DDM with collapsing bounds is optimal under certain assumptions (Tajima et al., 2016). However, we are not trying to show that our network model is solving the task optimally. The model's performance depends on the training dataset. Whether the training dataset reflects an optimal strategy or not, the network would exhibit the same. What we aim to show is that the network model is able to solve the task based on the experience. The experience comes from the training, and we postulate that a different system, most likely a goal-directed one, is responsible for the choice behavior during initial learning and teaches our network model. Our framework is analogous to the habitual system in the brain.

2.2 2) Page 20, lines 408-409: Authors' definition of Iwhen and Iwhich are very confusing to me. These measures don't seem to be doing what they are supposed to do. For example, if a unit has (WLT, WRT, WFT) respectively equal to (5, 2, 3.5), this unit is +which (5-2=3) but apparently not sensitive to fixation at all ((5+2)/2-3.5=0), which does not make sense. Why not pass them through a psychometric function and use their relative weights to compare units?

We think the reviewer's example unit actually supports our argument. As the reviewer correctly reasoned, the unit is classified as a +*which* unit by our definition and contributes to the leftward choice. However, notice that the task is a reaction-time task. Since (WLT+WRT)/2-WFT for this unit is zero, the unit does not, on average, contribute to the hold-versus-go decision. Therefore, this unit would be a nice example unit that dissociates the left/right decision from the hold-versus-go decision.

Once the -*when* units' activities increase and suppress the activity of the fixation unit, the left/right saccade units can trigger a choice depending on their relative activity levels, which depend on the hidden layer units that contribute to the left and right target differently, i.e. the *which* units.

2.3 3) Page 22, Figure 6: Can authors explain why the connection weights for +/- when and +/- which are not symmetric with respect to 0. Wouldn't it show that not only weights, but the activity of these units should be also considered?

Considering the fact that the network keeps fixation most of the time, it is reasonable that the connection weight of +/- *when* units are not symmetric with respect to 0. The connection weights are biased toward the positive side for the fixation and the negative side for the left/right saccade. Consistently, the units with -*I_when_* are more prevalent than the units with +*I_when_* (77.40 ± 0.89 v.s. 50.60 ± 0.89). When we plot the distribution of *I_when_* (Fig Supp2), we can see that the distribution of the -*I_when_* units has a longer tail than that of the +*I_when_* units.

The *I_which_* of the *when* units are unbiased to the left-verse-right choice, however. As shown in Fig Supp3, bottom row, the mean *I_which_* of + *when* and - *when* units are not significantly different from zero, suggesting the *when* units do not have a bias between the left and right decisions.

Fig Supp2. The *I_when_* of all units (blue) in all runs. Units selected as +*when* units and –*when* units are indicated by red and green, respectively. The lower panel shows the same distribution with only the *when* units at a finer vertical scale. The vertical black lines represent the mean value of each distribution.

Fig Supp3. a. The *I_when_* of +*which* units (left panel) and – *which* units (right panel) in all runs. b. The *I_which_* of +*when* units (left panel) and – *when* units (right panel) in all runs. Significance is measured with a t-test.

Similar arguments can be made for why +*/*- *which* units are not symmetric with respect to 0. The average connection weights between the *which* units and the left/right saccade units are smaller than the connection weights between the *which* units and the fixation unit, suggesting that when the activities of the *which* units are balanced, they on average favor holding the fixation. Indeed, when we calculate their *when* indices, on average they are negative (+*which:* -0.14±0.053, -*which:* -0.12±0.052), which indicates they contribute to holding decisions (Fig Supp 3, top row).

Our indices are a simple measure of how the units contribute to the choices. We may certainly adopt other ways to define these indices, including the one that the reviewer suggested. The results are largely consistent. We prefer to use our definition because it is simpler.

For example, we can define the indices that take account of the units' activities as follows:

$I_{when}^{'}=(\mu_{R_{left}}*w_{ho-LT}^{i}+\mu_{R_{right}}*w_{ho-RT}^{i})/2-\mu_{R_{fixation}}*w_{ho-FP}^{i}$

$I_{which}^{'}=\mu_{R_{left}}*w_{ho-LT}^{i}-\mu_{R_{right}}*w_{ho-RT}^{i}$

Here, $\mu_{R_{left}}$ and $\mu_{R_{right}}$ are the averaged activities of the unit at the time of the decision, while $\mu_{R_{fixation}}$ is the averaged activities of the unit during fixation. With these new definitions, we may select the +/-*when* and +/-*which* units as before. We find the +/-*when* and +/-*which* units selected with the new indices largely overlap with the ones selected with the old ones (Table 1). Finally, we plot Fig 6a again, but using the product between the connection weight and the units' activities (i.e. $\mu_{R_{fixation}}*w_{ho-FP}^{i}$, $\mu_{R_{left}}*w_{ho-LT}^{i}$, and $\mu_{R_{right}}*w_{ho-RT}^{i}$) instead of just the weight, as the reviewer suggested (Fig Supp4). The pattern is similar to Fig 6a, and the positive/negative units are still not symmetric with respect to 0. In the upper panel, we can see that the *when* units do not have a left/right bias. In the lower panel, the +*which* and -*which* units have similar outputs to the fixation. However, note that the activities we use to define these indices are at the time of decisions, so the average *connection weight*response* of the *which* units is now positive for the connections to the left/right saccade units.

| + *when* | - *when* | + *which* | - *which* |
| --- | --- | --- | --- |
| 0.759±0.0345 | 0.745±0.0285 | 0.643±0.0312 | 0.710±0.0442 |

Table 1. The proportion of units in each group using the new definition that belong to the same group based on the original definition.

Fig Supp4. The product of *when* (upper panel) / *which*( lower panel) units' responses and their connection weights to the eye movement output units.

2.4 4) Page 30, lines 624-627: I don't understand why only rewarded trails are used to train the network. What happens if all trials are fed into the network during the training? Unrewarded sequences carry same amount of information about the task as the rewarded trials.

We fully agree that the unrewarded trials may also play important roles during learning.

In the first three tasks, the network models are trained with datasets in which the choices are generated already with optimal algorithms. All choices in the training set are technically correct, even though sometimes a reward is not obtained due to the intrinsic uncertainty. Most of the choices lead to reward. Therefore, it actually does not matter whether the unrewarded trials are trained or not.

In the last task, however, the choices in the training set are generated randomly, and half of them lead to no rewards. If we feed all the trials into the network, the network would be trained to learn choice that lead to both rewards and no rewards. After the training, the network would learn which actions lead to rewards and which do not, but there are no mechanisms to help the network to choose between them.

When only the rewarded trials are used in training, the correct actions are more likely to be part of the training dataset than the wrong actions are. The network learns the correct choices because they are more likely to be found in the training dataset. Thus, during the testing phase, the predictions of the correct actions prevail.

We believe that the current model is analogous to the habitual system in the brain, which carries out actions that have been repeatedly rewarded. An alternative system, which we have not included in the current framework and is likely a goal-directed one, helps to train the habitual system by selectively using the rewarded trials.

It is an interesting question of how we can adapt the current framework to implement a selection mechanism based on the prediction of future rewards. This, however, is out of the scope of the current manuscript and will be one of our future investigation topics.

2.5 5) Page 32, line 671-673, & Page 37-39: Even though that the loss function of the trained network is not that of reinforcement learning, the network has access to the reward, its estimate of reward at each time point, and is required to match its actions to rewarding ones (necessary pieces for a reinforcement learning agent). So, what makes authors think that the network is not using some deviation of RL to solve the task? Recent studies have been focused on the relation of these two frameworks [1-2]. Authors cannot make such a strong claim considering the structure of their network.

The reviewer may have misunderstood our point. During the testing phase, the network is fixed, and there is no loss function or reward prediction error to be calculated, and no learning occurs in the sense of network plasticity. The decisions are based on network dynamics, which represent past events, including rewards. This is distinct from the biological implementation that has been proposed for RL, which is based on synaptic plasticity modulated with the reward prediction error.

The recommended papers show how the RL problem may be solved in the context of NLP and how NLP assists reinforcement learning. While they are interesting, we think we are discussing conceptually different things.

Seeing the confusion, we have revised our discussion to communicate these points better.

2.6 6) In all these experiments, only a single network is trained and used to calculate the average and SE values. How consistent are these results for different trained networks? What percentage of the trained networks show the mentioned behaviors?

We run 20 independent simulations for each task. Other than the example units, all the figures and the analyses are based on these 20 simulations. The error bars indicate the SE across runs. We have added the following sentence in the end of the *Network framework* section (line 233-234):

The results presented here are based on 20 independent runs for each task.

Minor concerns:

2.7 1) Page 3, line 48-49: There seems to be a few words missing/incorrect in this sentence;

"Therefore, we build a recurrent neural network framework based on the gated recurrent units and test how it matches experimental findings in four exemplar tasks, each focusing on a different aspect of decision making and learning."

We have rewritten this part of the abstract (Line 45-51) to make it clearer:

We wonder whether these neural networks, the gated recurrent unit (GRU) networks in particular, reflect how the brain solves the contingency problem. Therefore, we build a GRU network framework inspired by the statistical learning approach of NLP and test it with four exemplar behavior tasks previously used in empirical studies. The network models are trained to predict future events based on past events, both comprising sensory, action, and reward events.

2.8 2) Page 7, last paragraph: Please add a more detailed summary of your results in this section. The language is very vague for the introduction.

We have revised the final paragraph of the introduction as suggested (Line 134-143):

With four exemplary behavior tasks, we show that the network models are able to learn tasks with extended and variable length and make choices based on the contingencies between events far apart from each, including those across trials. The learning can be generalized to novel sequences and even novel conditions. Importantly, we show that the activity patterns of the network units resemble those of the neurons in the brain, which reflect accumulated evidence, choice, and value. These results, along with the similarities between our framework and the neural circuitries of decision making in the brain, suggest that the computational principle in the brain may be understood as a sequence learning process based on statistical inference.

2.9 3) Page 9, line 147: "Our network framework contains three layers …"

Fixed

2.10 4) I did not find any information on the criteria for training procedure. Was the training stopped after the error went below a certain threshold? If so, please add it to the related section.

The training stops after 7.5e5 trials. We used the same number of training trials for all four tasks. We have stated this number in the manuscript.

2.11 5) Please add a more detailed caption to Figures 4 and 11.

We have revised the captions as follows (Line 340-347, 716-721):

Figure 4. The performance of the network trained with a dataset that contains only 1000 unique sequences. Same conventions as in Fig 3. a. The psychometric curve. b. The leverage of each shape on choice. c. Reaction time distribution (bars, right y-axis) and the mean total logLR (lines, left y-axis) at the decision time. Green and red indicate the left and right choices, respectively. d. The leverage of the first 3, the second and third from the last, and the middle shapes on the choice. The error bars in all panels indicate SE across runs. Some error bars are smaller than the data points and not visible.

Figure 11. The network’s performance when the training dataset’s block size is 50 trials and the testing dataset’s block size is 70 trials. Same conventions as in Fig 10. a. The switching behavior. Trials are aligned to the block switch (trial 0). b. The probability of repeating the previous choice. c. Trial history effects. Solid dots indicate significant effect (Bonferroni correction, p < 0.05). e. Factors affecting the choices. ※ indicates significance (p<0.01).

2.12 6) Page 21, line 427: "The where and when group units only overlap rarely …"

Fixed

2.13 7) Page 21, line 429: Please report the prevalence of each group separately.

These numbers are provided (line 441-446).

2.14 8) Page 22, Figure 6a: Please add a horizontal line for 0 to both panels.

Fixed

2.15 9) Page 26, Figure 8b: Please add information on the error bars to the caption.

Fixed

2.16 10) Page 28, line 587: Please report the prevalence of these units in your model.

There are 89.2±1.20 out of 128 units that are selective to the choice during the delay period. We now state the number in the manuscript (Line 604).

References:

[1]. Luketina, J., Nardelli, N., Farquhar, G., Foerster, J., Andreas, J., Grefenstette, E., ... & Rocktäschel, T. (2019). A survey of reinforcement learning informed by natural language. arXiv preprint arXiv:1906.03926.

[2]. Jiang, Y., Gu, S. S., Murphy, K. P., & Finn, C. (2019). Language as an abstraction for hierarchical deep reinforcement learning. In Advances in Neural Information Processing Systems (pp. 9414-9426).
