# Supplementary material for: A recurrent neural network framework for flexible and adaptive decision making based on sequence learning
Source: PLoS Comput Biol. 2020 Nov 3;16(11):e1008342. doi: 10.1371/journal.pcbi.1008342 (PMC7673505; doi:10.1371/journal.pcbi.1008342)
Supplement: Attachment — Submitted filename: Response to reviewers.docx [file pcbi.1008342.s002.docx]

Reviewer's Responses to Questions

We would like to thank the referees for the all the help throughout the review process. Here are the responses to the remaining questions. Again, the referees' comments are quoted below in black; our responses are in blue.

**Comments to the Authors:**

**Please note here if the review is uploaded as an attachment.**

Reviewer #1: The authors have done a reasonable job in addressing my concerns.

1.1 Fig 1a should read "Pavlovian"

Fixed.

Reviewer #2: I would like to thank the authors for responding to my questions. I have a recommendation to add:

2.1 RE 2.3 and 2.4: I suggest the authors to add a few sentences to the main manuscript explaining these points.

As recommended, we add the following sentences to help readers to understand our work.

Line 284-285

Only the events in the rewarded trials are used in training, but the results hold if all trials are used, since most choices generated with the DDM lead to rewards.

Line 657-661

If we include both rewarded and unrewarded trials during training, the network would learn choices that lead to both rewards and no rewards. Although the reward outcome of a particular action is predictable, there are no a mechanism in the current model to choose between the actions based on this prediction of rewards, and the network would only generate random choices.

Line 451-456

The connection weights of the both when groups units are biased toward the positive side for the fixation and the negative side for the left/right saccade (Fig 6a), because the network keeps fixation most of the time. Similarly, the average connection weights between the which units and the left/right saccade units are also negative (Fig 6a), suggesting that the which units are also slightly biased toward holding the fixation.
